# The Impact of Cardiovascular Health and Welfare on Meat Quality in Modern Broiler Chickens: An Integrated Approach to Poultry Production

**DOI:** 10.3390/ani16182959

**Published:** 2026-09-20

**Authors:** Wiktoria Kościów, Emilia Lisiecka, Monika Łukasiewicz Mierzejewska

**Affiliations:** Institute of Animal Science, Warsaw University of Life Sciences, Ciszewskiego 8, 02-786 Warsaw, Poland; wiktoria_kosciow@sggw.edu.pl (W.K.); emilia_lisiecka@sggw.edu.pl (E.L.)

**Keywords:** broiler chickens, cardiovascular health, animal welfare, breast muscle myopathies, hypoxia, microcirculation, endothelial dysfunction, meat quality

## Abstract

Modern broiler chickens have been selected for very rapid growth, efficient feed use, and a high proportion of breast muscle. However, the development of the cardiovascular and respiratory systems may not always keep pace with the increasing metabolic demands of rapidly growing tissues. This imbalance may contribute to reduced blood flow, insufficient oxygen delivery, oxidative stress, inflammation, and abnormal muscle regeneration. At the same time, environmental and management factors such as heat stress, high stocking density, poor air quality, unsuitable lighting programs, limited activity, and pre-slaughter stress can further challenge the birds’ ability to maintain normal physiological function. This review explains how cardiovascular health and welfare conditions may interact in the development of breast muscle abnormalities and changes in meat quality. Particular attention is given to impaired circulation, tissue oxygen deficiency, vascular dysfunction, and their possible links with conditions such as wooden breast, white striping, and spaghetti meat. Understanding these relationships may help improve broiler health and welfare while maintaining meat quality and production efficiency. Future research should combine measurements of cardiovascular function, bird behaviour, environmental conditions, muscle health, and meat quality in the same animals over time.

## Simple Summary

Modern broiler chickens have been selected for rapid growth and high breast muscle yield, which increases their demand for oxygen and nutrients. However, the cardiovascular and respiratory systems may not always develop sufficiently to meet these demands. This may contribute to reduced blood flow, tissue oxygen deficiency, oxidative stress, inflammation, and breast muscle abnormalities such as wooden breast, white striping, and spaghetti meat. Welfare-related factors, including heat stress, stocking density, lighting, limited activity, poor air quality, transport, and pre-slaughter stress, may further increase physiological strain. This review examines how cardiovascular health and welfare conditions may interact in the development of muscle abnormalities and changes in meat quality. A better understanding of these relationships may help improve broiler welfare, health, production efficiency, and the quality of poultry meat.

## 1. Introduction

Modern broiler chickens represent an outstanding example of highly effective selection for rapid growth, low feed consumption, and an increased proportion of breast muscle in the carcass [1,2,3,4]. However, advances in production traits have not always been accompanied by proportional adaptation of the cardiovascular, respiratory, and musculoskeletal systems. Consequently, the rapidly growing bird operates under a high demand for oxygen and nutrients, while the capacity of the circulation to deliver them may be limited. This mismatch may contribute to health issues such asmetabolic disorders, pulmonary hypertension, ascites, structural abnormalities of the breast muscles, and sudden death of the bird. In this context, meat defects should not be regarded solely as technological problems. They are suspected to represent the end-stage manifestation of chronic metabolic overload, inadequate perfusion, hypoxia, and impaired muscle regeneration. At the same time, not every alteration in meat quality is evidence of compromised welfare; therefore, causal relationships must be distinguished from the co-occurrence of traits arising from a shared genetic and physiological background [5,6,7,8].

Over the past several decades, the growth rate of broiler chickens has increased dramatically, accompanied by greater breast muscle yield and improved feed efficiency. Comparisons between historical and modern broiler lines show that body weights requiring a much longer rearing period are now attained within only a few weeks, illustrating the magnitude of selection pressure for growth and meat yield [9,10,11]. Such rapid phenotypic change raises the question of whether the development of the cardiovascular system, pulmonary circulation, and muscle microvasculature have progressed proportionally to the increasing metabolic demands of the tissues.

Despite the growing body of research on wooden breast, white striping, and spaghetti meat, the mechanisms linking intensive growth, cardiovascular health, welfare, and meat quality remain incompletely understood. Previous studies have frequently focused on individual components of this system—pulmonary hypertension, muscle hypoxia, mitochondrial dysfunction, oxidative stress, or meat quality alterations—rather than examining them as an interconnected pathophysiological continuum. Increasing evidence suggests that impaired perfusion, insufficient capillarization, endothelial dysfunction, and disturbances in the hypoxic response may constitute important links between breast muscle hypertrophy, degeneration, and defective regeneration [3,4,7,8,12,13,14].

This review therefore aims to critically evaluate the hypothesis that cardiovascular and microcirculatory dysfunction represents a mechanistic link between intensive selection in modern broilers and subsequent changes in welfare and meat quality. Particular attention is given to the relationships between rapid growth, cardiovascular and microcirculatory limitations, breast muscle myopathies, and their consequences for meat quality. The review also considers the modifying role of welfare-related factors and evaluates the potential of breast myopathies and selected biomarkers as indicators of chronic physiological imbalance and increased susceptibility to muscle disorders.

## 2. Methodology

This narrative review was based on a structured search of the scientific literature conducted using the PubMed, Scopus, Web of Science, and Google Scholar databases. The search focused primarily on peer-reviewed articles published between 2000 and 2026, although earlier seminal studies were also included when they provided essential information on the development of pulmonary hypertension, ascites syndrome, deep pectoral myopathy, or the historical evolution of broiler growth and selection.

The principal search terms included combinations of the following keywords: broiler chickens, cardiovascular function, pulmonary hypertension, ascites, right ventricular hypertrophy, microcirculation, capillary density, muscle perfusion, hypoxia, HIF-1α, VEGF, endothelial dysfunction, oxidative stress, wooden breast, white striping, spaghetti meat, breast muscle myopathies, welfare, heat stress, stocking density, lighting, transport stress, nutrition, arginine, guanidinoacetic acid, phytase, antioxidants, meat quality, pH, water-holding capacity, cooking loss, and shear force. Boolean operators (“AND”, “OR”) were used to combine terms from different thematic areas.

Priority was given to original experimental studies, comparative studies, and recent review articles directly addressing relationships among growth rate, cardiovascular or microvascular function, breast muscle pathology, welfare-related factors, and meat-quality traits. Studies were considered particularly relevant when they provided quantitative data on physiological, histological, biochemical, or meat-quality outcomes. Articles were excluded when they were not related to broiler chickens, lacked sufficient methodological detail, focused exclusively on unrelated poultry species, or did not contribute directly to the mechanistic relationships evaluated in this review. However, selected studies in other avian species or earlier foundational studies were retained when they provided important physiological or pathological context.

The literature was assessed qualitatively with particular attention to the strength and directness of evidence. Findings derived from longitudinal or experimental studies were distinguished from cross-sectional associations and mechanistically plausible hypotheses inferred from separate studies. Conflicting results were interpreted in relation to differences in genotype, age, slaughter weight, environmental conditions, severity of myopathy, tissue sampling location, and analytical methodology. Because the review was narrative rather than systematic, no formal meta-analysis or quantitative risk-of-bias assessment was performed.

**Figure 1:**
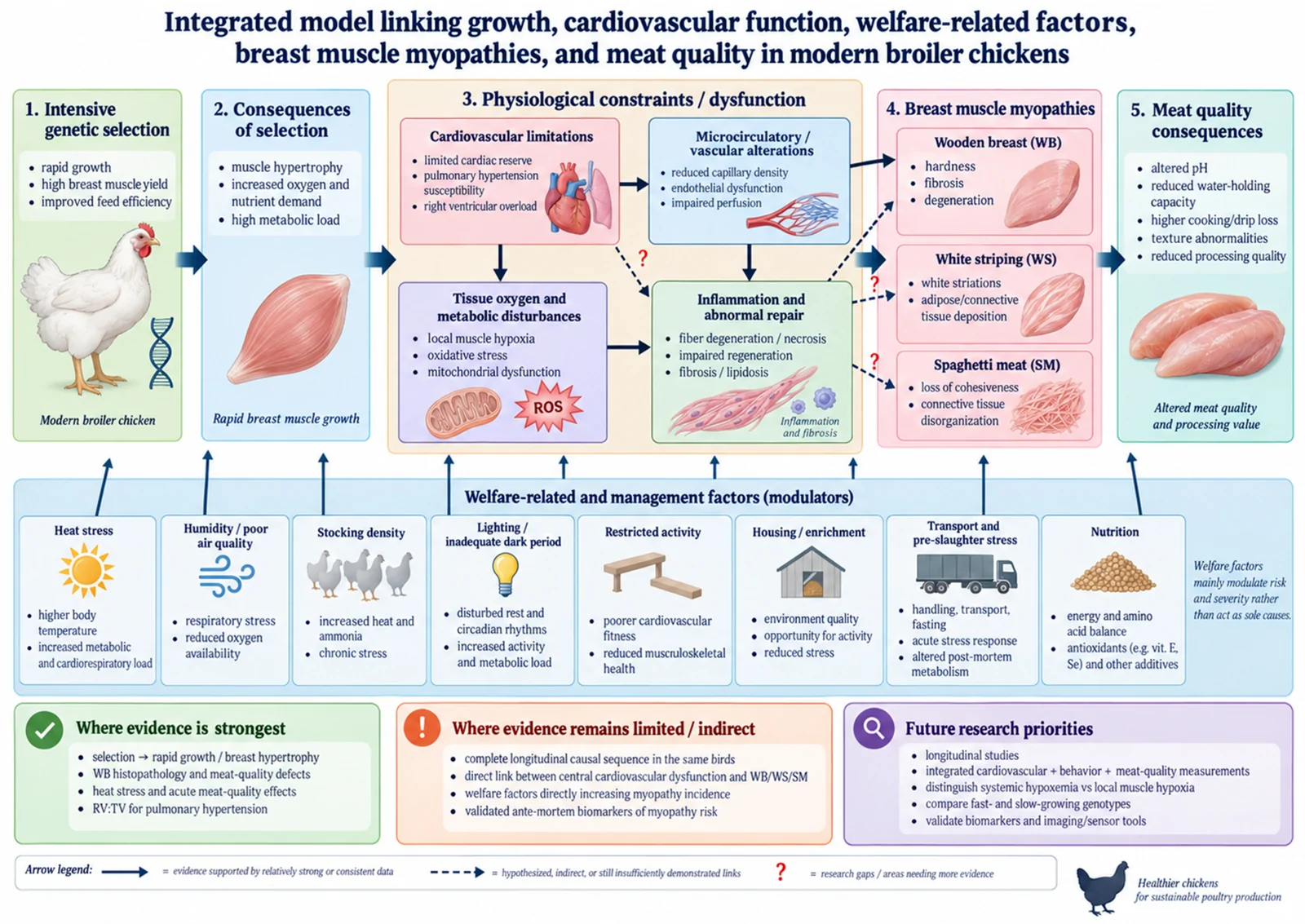
Integrated_model linking growth, cardiovascular function, welfare-related factors, breast muscle myopathies, and meat quality in modern broiler chickens.

Figure 1 was created with the assistance of a generative artificial intelligence tool. The tool was used solely to generate a schematic graphical representation illustrating the relationships described in the manuscript. The generated figure was subsequently reviewed, edited, and verified by the authors to ensure its scientific accuracy and consistency with the content of the manuscript. No AI-generated content was used as a source of scientific data or evidence.

## 3. Genetic Selection and Development of the Circulatory System

Selection of broilers for high body weight, high breast muscle yield, and improved feed efficiency has altered not only their production phenotype but also the relationships among major physiological systems. Increasing muscle mass raises oxygen demand, requires greater blood flow, and depends on effective adaptation of the heart and vasculature. In birds susceptible to pulmonary hypertension, the anatomical capacity of the pulmonary vasculature may be insufficient to accommodate the increasing cardiac output, resulting in increased vascular resistance and right ventricular overload [6]. At the same time, hypertrophy of breast muscle fibers is not always accompanied by proportional capillary development [2,13,15]. However, these observations should not be interpreted as evidence that rapid growth inevitably causes cardiovascular or microcirculatory failure.

The magnitude of selection-driven change is illustrated by historical genotype comparisons. Havenstein et al. [9,10] reported that at 42 days of age a commercial broiler representing the 2001 genotype weighed approximately 2.67 kg, compared with only about 0.54 kg in the 1957 control line. The modern genotype reached approximately 1.8 kg at 32 days, whereas the historical line required more than 100 days to attain a comparable weight. Zuidhof et al. [11] subsequently confirmed that growth rate increased by more than 400% between 1957 and 2005, while feed conversion improved by approximately 50%. Over the same period, the relative contribution of the *Pectoralis major* to carcass weight increased by approximately 79% in males and 85% in females. These data demonstrate the exceptional magnitude of genetic progress, but they do not by themselves prove proportional underdevelopment of the cardiovascular system. Rather, they document the increase in tissue growth and metabolic demand against which cardiovascular and microcirculatory adaptation must be evaluated.

Direct comparisons among broiler genotypes indicate that growth rate and breast yield are related, but not interchangeable, determinants of myopathy susceptibility. Santos et al. [16] evaluated 7216 birds representing 14 strains, including two conventional strains with average daily gain above 60 g/day and 12 slower-growing strains ranging from <50 to 55 g/day. At comparable target body weights of approximately 2.1 and 3.2 kg, breast yield increased significantly with growth rate (*p* < 0.001), and conventional strains generally exhibited the highest incidence of wooden breast and white striping at the heavier slaughter weight (*p* < 0.001). Importantly, however, one of the faster-growing strains within the slower-growing category showed myopathy incidence comparable to, or even greater than, that of conventional birds. This indicates that high breast yield, rather than growth rate alone, may be a particularly important determinant of myopathy risk.

Genetic analyses further challenge a simple deterministic interpretation. Bailey et al. [17] reported heritabilities of major breast muscle myopathies ranging only from 0.04 to 0.25, while genetic correlations with body weight and breast yield ranged from −0.06 to 0.41. Phenotypic correlations were similarly low to moderate (0.02–0.23), and more than 71.5% of the phenotypic variance was attributed to residual, non-genetic effects. Moreover, a broiler population representing approximately two additional years of genetic progress showed an 18.4% relative reduction in wooden breast incidence together with a 1.02% relative increase in breast yield, demonstrating that improvement in production traits does not necessarily require a parallel increase in myopathy prevalence [17].

Taken together, these findings argue against treating breast myopathies as an unavoidable consequence of selection for rapid growth. Instead, the available evidence supports a multifactorial model in which growth rate, breast muscle yield, genetic susceptibility, microvascular development, nutrition, and environmental conditions interact to determine risk. Importantly, direct genotype comparisons provide considerably stronger evidence for an association between growth-related traits and myopathy incidence than for a causal link between these traits and cardiovascular dysfunction. Thus, while disproportionate muscle growth remains biologically plausible as a contributor to impaired tissue perfusion, the extent to which central cardiovascular limitation is a primary driver of WB, WS, or SM remains insufficiently demonstrated and should be interpreted cautiously.

## 4. Anatomy and Physiology of the Broiler Cardiovascular System

The avian cardiovascular system supports a high metabolic rate, transports oxygen and energy substrates, and removes heat and metabolic by-products. The four-chambered heart ensures complete separation of oxygenated and deoxygenated blood; however, overall system efficiency also depends on pulmonary vascular resistance, vascular bed capacity, and microcirculatory function. The endothelium is of particular importance because it regulates vascular tone, permeability, inflammation, coagulation, and angiogenesis. In the breast muscle, capillaries determine the availability of oxygen, glucose, and amino acids and facilitate the removal of lactate and other metabolites. Nevertheless, high blood flow does not guarantee adequate tissue oxygenation when the vascular network is insufficiently dense or endothelial alterations are present. Accordingly, broiler physiology should be evaluated by considering cardiac function, pulmonary circulation, peripheral microcirculation, and the ability of muscle tissue to utilize delivered oxygen as an integrated system rather than as isolated components [4,6,13,14].

## 5. Disproportion Between Muscle Growth and Vascularization

Growth of the broiler breast muscle occurs predominantly through hypertrophy of existing fibers, accompanied by an increase in fiber diameter and a reduction in the space available for vessels within the endomysium and perimysium. If angiogenesis fails to keep pace with tissue growth, the number of capillaries per unit fiber area decreases and the diffusion distance for oxygen from blood to the interior of the cell increases. The superficial and cranial regions of the breast muscle may be particularly vulnerable, as these are often among the first areas in which changes characteristic of wooden breast are detected. Insufficient capillarization restricts not only oxygen delivery but also metabolite removal and regulation of the ionic microenvironment. Interpretation should nevertheless remain cautious: reduced vascular density may be a cause of injury, a consequence of remodeling in diseased tissue, or both. The most plausible model is therefore one in which hypertrophy, insufficient angiogenesis, mechanical compression, and abnormal regeneration mutually reinforce one another [2,3,7,13,14,15,18,19]. Hypoxia may also affect non-myofibre cell populations involved in muscle maintenance and regeneration. In particular, satellite cells are sensitive to oxygen availability, and experimental evidence indicates that hypoxic conditions can alter their proliferation and impair myogenic differentiation [20]. Fibro-adipogenic progenitor (FAP) cells may also contribute to pathological remodeling by promoting fibrosis and adipose deposition when regenerative signaling becomes dysregulated, although their specific response to hypoxia in broiler breast muscle remains poorly characterized. Thus, hypoxia-related dysfunction may involve not only mature muscle fibres but also the cellular populations responsible for regeneration and extracellular-matrix remodeling.

An important physiological limitation of the hypoxia hypothesis is that the broiler *Pectoralis major* is composed predominantly of fast-twitch glycolytic fibers with relatively low mitochondrial density and a strong dependence on anaerobic glycolysis. Consequently, its response to reduced oxygen availability may differ fundamentally from that of more oxidative muscles. Hypoxia should therefore not be interpreted as acting on *Pectoralis major* in the same manner as in highly oxidative skeletal muscle, and some hypoxia-related molecular or structural changes may reflect secondary responses to established myopathy rather than primary initiating events [7,18,19]. Direct comparisons between affected and unaffected breast muscle and more oxidative locomotor muscles remain scarce, which currently limits conclusions as to whether the observed hypoxia-related responses are specific to *Pectoralis major* or reflect more general muscle pathology.

Available experimental data provide partial support for this model, although they do not establish a complete causal sequence. Sihvo et al. [13] compared unaffected broilers (*n* = 14) with birds showing focal WB lesions (*n* = 14) and found that the transverse myofibre area supplied by a single vessel was significantly greater in macroscopically unaffected muscle adjacent to WB lesions than in unaffected control muscle (*p* = 0.01), indicating a relative reduction in vascular supply already at an early stage of the disorder. Ultrastructural examination further revealed mitochondrial hyperplasia and enlargement of the sarcoplasmic reticulum in minimally affected fibres, changes compatible with altered oxygen supply or osmotic imbalance [13]. Importantly, Papah et al. [14] demonstrated in a longitudinal study that vascular pathology preceded advanced muscle degeneration: localized phlebitis and lipogranulomas were detectable as early as week 1, focal myofibrillar degeneration appeared in week 2, whereas overt inflammatory infiltration became evident from week 3 and extensive degeneration, necrosis and fibrosis developed between weeks 4 and 7. Similarly, Griffin et al. [15], following commercial broilers from 2 to 46 days of age (*n* = 27 per sampling day), showed that increasing *Pectoralis major* depth and breast muscle yield were associated with progression toward more severe myopathy stages. However, these studies were conducted in fast-growing, high-yielding commercial populations and did not directly manipulate vascular supply or compare matched fast- and slow-growing genotypes. Therefore, they support an association between rapid muscle hypertrophy, reduced relative vascularization and early pathological change, but do not prove that insufficient angiogenesis is the primary initiating cause of WB. Reduced vessel density could still represent both a predisposing factor and a consequence of tissue remodeling, and this distinction remains unresolved.

## 6. Tissue Hypoxia as a Key Pathological Mechanism

Hypoxia is one of the principal mechanisms proposed in the pathogenesis of breast muscle myopathies. Reduced oxygen availability stabilizes HIF-1α and activates an adaptive response aimed at maintaining cell survival [1,7,19,21]. This response involves increased anaerobic glycolysis, reduced mitochondrial substrate oxidation, and changes in glucose and lipid metabolism. Short-term activation may be beneficial, whereas chronic hypoxia promotes reduced efficiency of ATP production, lactate accumulation, mitochondrial dysfunction, and increased generation of reactive oxygen species [22]. The HIF-1 pathway may also interact with mechanisms of fibrosis and lipid deposition, thereby contributing to features typical of wooden breast and white striping [7,19]. Experimental and omics-based studies support this association, but the evidence remains indirect: Abasht et al. [21] identified marked metabolic disturbances and oxidative stress in WB-affected muscle, while Wang et al. [22] reported 3444 differentially expressed genes and 119 altered metabolites, with prominent disruption of mitochondrial and energy–metabolism pathways. However, these studies compared affected and unaffected tissue after pathological changes had already developed and therefore cannot establish whether hypoxia is an initiating event or a secondary consequence of muscle degeneration. Importantly, Jung et al. [20] demonstrated experimentally that hypoxia directly alters broiler satellite-cell behavior by promoting proliferation and inhibiting myogenic differentiation, providing mechanistic support for impaired regeneration under low-oxygen conditions; however, this in vitro evidence does not demonstrate that hypoxia is the primary cause of WB in vivo. However, not all studies support a unidirectional activation of angiogenesis. Reduced VEGFA expression has been reported in severely affected muscles, which may indicate an insufficient or exhausted adaptive response. Hypoxia should therefore be regarded as one component of a pathological network rather than as the single and exclusive cause of these myopathies [7,20].

## 7. Endothelial Dysfunction and Vascular Alterations

Endothelial dysfunction may represent an important, although still insufficiently understood, link between rapid muscle growth and tissue injury. In wooden breast-affected muscle, phlebitis involving small veins and venules, perivascular lesions, thickening of endothelial cells, and an abundance of organelles indicative of metabolic activation have been described [3,4,14]. A damaged or excessively activated endothelium may be less effective in regulating vascular tone, permeability, leukocyte migration, and extracellular matrix remodeling. Increased permeability promotes edema, which raises intramuscular pressure and may further restrict blood flow. Oxidative stress, in turn, reduces nitric oxide bioavailability and enhances inflammatory responses. It remains unresolved whether endothelial dysfunction initiates muscle injury or occurs secondarily to necrosis and inflammation. A feedback mechanism is most plausible: vascular abnormalities impair perfusion, whereas damaged tissue releases mediators that further activate the endothelium [4,7].

## 8. The Heart and Central Circulatory Failure

Pulmonary hypertension and ascites are among the best-characterized cardiorespiratory disorders of fast-growing broilers. They develop when increasing cardiac output exceeds the flow capacity of the pulmonary vascular bed. Elevated pulmonary arterial resistance increases pulmonary pressure, overloading the right ventricle and potentially leading to further conditions such as right ventricular hypertrophy, atrioventricular valve insufficiency, venous congestion, and fluid accumulation within the abdominal cavity [6]. Clinical ascites, however, represents an end-stage manifestation. Some birds may exhibit milder or subclinical abnormalities, including elevated pulmonary pressure, limited cardiac reserve, or inadequate blood oxygenation in the absence of overt clinical signs. Quantitative studies support the existence of substantial cardiac dysfunction before or alongside overt ascites. Olkowski et al. [23] reported that left ventricular fractional shortening was reduced to 21.7 ± 2.0% in ascitic broilers, compared with 39.1 ± 3.6% in unaffected broilers and 43.3 ± 2.4% in Leghorns (*p* < 0.01); Doppler examination also revealed atrioventricular valve regurgitation in ascitic birds and in some apparently normal broilers. These findings indicate that functional cardiac abnormalities can precede obvious clinical manifestations, but they do not demonstrate that such dysfunction directly causes breast myopathies or deterioration in meat quality. Such conditions may reduce the ability of the organism to respond to physical exertion, high ambient temperature, or pre-slaughter stress. Nevertheless, evidence is currently insufficient to directly attribute every case of impaired meat quality to central cardiac failure; this relationship requires studies in which the same individuals are evaluated both before and after slaughter [6].

## 9. The Respiratory System as a Component of the Oxygen Transport System

The broiler respiratory system should be considered an integral component of the physiological system responsible for oxygen uptake, transport, and utilization by rapidly growing tissues. Its efficiency depends not only on ventilation and pulmonary gas exchange, but also on cardiac performance, blood characteristics, vascular flow, and muscle capillary density. In modern broilers, high metabolic demand may exceed the adaptive capacity of the cardiorespiratory system, particularly under adverse microclimatic conditions. High ambient temperature intensifies panting, causing excessive carbon dioxide loss, acid–base disturbances, and respiratory alkalosis [24,25]. High relative humidity, in turn, reduces the efficiency of evaporative heat loss, which increases respiratory effort and may exacerbate heat stress [26,27]. The source material further indicates that high humidity promotes deterioration of litter quality and increased ammonia concentrations, which damage respiratory mucous membranes and increase susceptibility to infection.

However, hypoxemia, defined as inadequate oxygenation of the blood, must be distinguished from local muscle hypoxia [7,13]. Muscle tissue may remain hypoxic even when blood oxygen saturation is relatively normal if microcirculatory disturbances, endothelial dysfunction, reduced capillary density, or an increased oxygen diffusion distance are present. Thus, air quality, ventilation, temperature, and humidity may increase the burden on the entire oxygen transport system, but they are not the sole determinants of hypoxia in the breast muscle. Their effects should be interpreted in relation to genotype, growth rate, cardiac function, and the degree of tissue vascularization.

## 10. Oxidative Stress and Inflammation

Oxidative stress is a potential mediator of muscle injury associated with impaired perfusion and hypoxia, but it should not be regarded as a specific marker of cardiovascular dysfunction. Restricted oxygen supply disrupts mitochondrial function and may increase the production of reactive oxygen species, particularly during episodes of ischemia and reperfusion. Excess free radicals promote lipid peroxidation, protein oxidation, membrane damage, and disruption of calcium homeostasis. These alterations lead to degeneration and necrosis of muscle fibers, which initiate the recruitment of inflammatory cells. Heterophils and macrophages contribute to removal of damaged structures but may simultaneously intensify oxidative stress through the release of cytokines and additional reactive oxygen species. Under normal conditions, the inflammatory response should culminate in regeneration; in myopathic muscles, however, repeated cycles of injury, incomplete repair, and fibrosis are observed [1,3,7,21,22,28,29].

Evidence from WB studies supports the presence of oxidative and metabolic disturbances, but does not establish their cardiovascular origin. Abasht et al. [21] demonstrated marked oxidative stress and metabolic perturbations in WB-affected muscle, whereas subsequent transcriptomic and metabolomic analyses identified extensive disruption of mitochondrial and energy–metabolism pathways [22]. Because these studies evaluated muscle with established or developing pathology, oxidative stress could represent both a contributor to tissue injury and a downstream consequence of mitochondrial dysfunction, inflammation, or abnormal regeneration. Thus, its presence alone cannot distinguish cardiovascular or microcirculatory impairment from other physiological challenges. The studies currently cited in the manuscript support oxidative/metabolic disturbances in WB, but not a unique cardiovascular source for them.

The severity of oxidative processes is also influenced by welfare and environmental conditions. Near-continuous lighting disrupts melatonin rhythms and can alter antioxidant status and nonspecific immunity; broilers exposed to different lighting regimens have shown measurable differences in antioxidant and immune parameters [30,31]. Oxidative stress can also arise from heat exposure, nutritional imbalance, infection, transport stress, and primary mitochondrial dysfunction. Consequently, oxidative-stress markers should be interpreted as nonspecific indicators of cellular challenge rather than direct indicators of cardiovascular failure and should be evaluated together with measurements of perfusion, systemic oxygenation, histopathology, HIF-1α-related signaling, and inflammatory parameters.

## 11. Circulatory Alterations and Breast Muscle Myopathies

Growth-related breast muscle myopathies comprise phenotypically and pathophysiologically distinct abnormalities that frequently coexist but should not be considered interchangeable manifestations of a single disease process. Wooden breast (WB) is predominantly characterized by increased muscle hardness, myofiber degeneration and necrosis, inflammation, vascular alterations, lipidosis, and extensive fibrosis. White striping (WS) is primarily manifested by white intramuscular striations associated with myofiber degeneration and increased adipose and connective-tissue deposition. In contrast, spaghetti meat (SM) is distinguished by loss of structural cohesiveness and impaired organization of the extracellular matrix and connective tissue. The principal differences among these abnormalities, including representative quantitative effects on meat-quality traits, are summarized in Table 1.

Importantly, the strength of evidence supporting vascular involvement differs among the three conditions. Vascular and endothelial abnormalities have been extensively described in WB, although their temporal relationship with muscle degeneration remains unresolved. For WS, the association with rapid muscle growth and breast yield is well established, whereas evidence for a primary vascular mechanism is less direct. SM appears to be particularly associated with altered connective-tissue development and collagen organization. Thus, impaired perfusion and tissue hypoxia may represent components of a broader pathogenic network rather than a single mechanism uniformly explaining all breast muscle myopathies.

Deep pectoral myopathy (DPM) is a well-established ischemic condition in which exercise-induced enlargement of the *supracoracoideus* within a relatively non-compliant osteofascial compartment increases intramuscular pressure, compresses the vascular supply, and results in ischemic necrosis [41,42]. Experimental studies have demonstrated that ischemia develops rapidly after stimulation of the *supracoracoideus* and can be prevented by surgical release of the overlying fascia [41]. In contrast, the superficial *Pectoralis major* is not enclosed within an equivalent restrictive compartment and was not affected under comparable experimental stimulation; therefore, the mechanism of DPM should not be directly extrapolated to WB, WS, or SM [43].

## 12. Effects of Circulatory Dysfunction on Meat Quality Traits

Breast muscle myopathies affect technological meat quality to different extents depending on the type and severity of the lesion. Among the major abnormalities, wooden breast (WB) generally produces the most pronounced deterioration in processing properties and is frequently associated with higher ultimate pH, increased drip and cooking losses, reduced water-holding capacity, and abnormal hardness or toughness [2,33,38,44,45]. In the current manuscript, these effects were already linked to higher pH, greater storage and cooking losses, reduced water-holding capacity, and altered toughness. White striping (WS) usually exerts a more moderate and severity-dependent effect, with changes most consistently reported for water binding, marinade uptake, cooking yield, and chemical composition [33,37,38]. In contrast, spaghetti meat (SM) is primarily characterized by impaired structural cohesiveness and altered connective-tissue organization, resulting in a softer, less compact muscle structure rather than the increased hardness typical of WB [33,39,40]. Representative quantitative outcomes and direct comparisons among WB, WS, and SM are summarized in Table 1.

Cross-study comparisons should nevertheless be interpreted cautiously because the magnitude of quality deterioration is strongly influenced by genotype, age and slaughter weight, lesion severity, anatomical sampling site, coexistence of multiple myopathies, and analytical methodology [2,33,44,45]. In addition, some quality traits may be modified by pre-slaughter and post-mortem factors independently of chronic myopathy development, including transport conditions and chilling procedures [45,46,47]. Therefore, differences between affected and unaffected breast meat should be interpreted as study-specific effect estimates rather than fixed diagnostic values.

## 13. Welfare as a Factor Exacerbating Cardiovascular Dysfunction

Housing and management conditions may act as important modifiers of the intrinsic susceptibility of broiler chickens to cardiovascular disorders. It is important to distinguish animal welfare as the individual bird’s physical and mental state from welfare-related environmental and management factors, such as temperature, humidity, stocking density, lighting, air quality, and handling, which can influence that state but are not themselves welfare outcomes. In fast-growing birds, the circulatory system already functions under considerable metabolic demand, which can reduce physiological resilience to suboptimal environmental conditions. Consequently, even moderate disturbances in housing conditions may increase cardiorespiratory strain, compromise tissue oxygen delivery, and promote oxidative imbalance. Rather than acting as isolated external variables, welfare-related factors can therefore modify the physiological responses that influence both muscle function and subsequent post-mortem metabolism. The magnitude of these effects is determined by the interaction between the nature and duration of the challenge and the bird’s age, genotype, and capacity for adaptation. The simultaneous occurrence of several challenges—for example, high temperature, high humidity, and high stocking density—is particularly unfavorable because their effects may be mutually reinforcing [5,25,27].

Lighting programs illustrate the difficulty of separating direct cardiovascular effects from indirect welfare-related consequences. Photoperiods exceeding 20 h/day have been associated with increased mortality from metabolic, cardiac, and skeletal disorders, whereas longer dark periods have been linked with fewer leg problems [48,49,50]. Darkness also supports melatonin secretion, circadian synchronization, and uninterrupted rest, while near-continuous lighting can fragment sleep and alter antioxidant capacity [30,50]. However, available studies do not demonstrate that lighting directly induces cardiovascular dysfunction or breast myopathies. Its effects are more plausibly mediated through changes in activity, growth rate, rest, oxidative status, and metabolic demand; photoperiod should therefore be regarded as a risk-modifying factor rather than an independent causal determinant.

Heat stress provides stronger evidence for a direct physiological challenge. Birds dissipate heat primarily through peripheral vasodilation and panting, which increases respiratory effort, alters blood-flow distribution, and promotes respiratory alkalosis and electrolyte imbalance [24,25]. Prolonged heat exposure also increases oxidative stress, impairs mitochondrial function, reduces feed intake, and modifies muscle energy metabolism [51,52,53]. These changes can influence post-mortem pH decline, water-holding capacity, colour, and shelf life. Importantly, acute and chronic heat exposure should not be treated as equivalent: acute pre-slaughter stress primarily affects glycogen use and post-mortem glycolysis, whereas chronic exposure involves both adaptive responses and prolonged oxidative and circulatory load. The magnitude of the response is also genotype-dependent, which limits direct extrapolation between experimental models and commercial strains.

Humidity acts mainly as a context-dependent modifier rather than an isolated cause. High relative humidity reduces evaporative heat loss and increases panting, thereby increasing cardiorespiratory demand [26]. Its adverse effects are amplified when high temperature is present, while prolonged humidity also worsens litter quality and promotes ammonia release. Ammonia exposure damages the respiratory tract and may impair gas exchange, whereas wet litter increases the risk of footpad dermatitis and breast skin lesions, with subsequent effects on activity [27,54]. Because humidity is closely linked with ventilation, stocking density, drinking systems, litter type, and temperature, its independent contribution is difficult to quantify and should be interpreted within the overall microclimatic environment.

A similar problem applies to stocking density. High stocking density restricts movement and species-specific behaviour and often coincides with increased temperature, humidity, and airborne contaminants [5,55,56]. It can therefore influence the cardiovascular system indirectly through heat load, poorer air quality, reduced locomotor activity, and impaired leg health [57,58]. Disturbed rest due to frequent contact with other birds provides an additional pathway [59]. However, the evidence is not uniform: in studies where ventilation, temperature, and feed access were adequately controlled, the effects of stocking density were reduced. This suggests that at least part of the observed response is attributable to associated deterioration of the microclimate rather than to bird density itself.

Housing system and environmental enrichment should also be interpreted cautiously. Enrichment and outdoor access can increase activity and behavioural opportunities and may improve leg and bone condition [60], but they do not automatically guarantee better welfare or meat quality. Outdoor systems also expose birds to greater climatic variability, parasites, and environmental pathogens. Moreover, the benefits are strongly genotype-dependent, because fast-growing broilers may use outdoor areas less extensively than slower-growing strains [61,62,63]. Consequently, production systems should be assessed using multiple outcomes, including behaviour, health, mortality, locomotor activity, cardiovascular indicators, and meat quality, rather than being classified as intrinsically beneficial or detrimental on the basis of system type alone.

Transport and pre-slaughter handling represent a different category because they cause acute physiological responses rather than chronic rearing effects. Catching, crating, loading, transport, and lairage increase physical activity, body temperature, oxygen demand, and glycogen utilization, while prolonged immobilization can impair ventilation and muscle blood flow [46,47]. Their impact depends on transport duration, temperature, humidity, crate density, ventilation, and the initial health status of the birds. Individuals with limited cardiac reserve or subclinical pulmonary hypertension are likely to tolerate such challenges less effectively. Acute pre-slaughter stress can directly alter muscle metabolism, pH decline, colour, and water-holding capacity; therefore, favourable rearing conditions do not necessarily ensure high meat quality if transport and slaughter conditions are suboptimal [47,51].

Overall, welfare-related factors should be interpreted as modulators of genetic and physiological susceptibility rather than as direct causes of breast muscle myopathies. They influence oxygen demand, cardiac workload, blood-flow distribution, acid–base balance, activity, and oxidative status, but the strength of evidence differs substantially among exposures. Heat stress and pre-slaughter stress have relatively well-established acute physiological and meat-quality effects, whereas the direct links between lighting, humidity, stocking density, enrichment, or reduced activity and WB, WS, or SM remain less well demonstrated. Representative studies examining these welfare-related factors, together with their main physiological and meat-quality outcomes and the strength of the available evidence, are summarized in Table 2. A major limitation is that most studies evaluate these factors separately, although commercial broilers are exposed to several challenges simultaneously. Studies integrating environmental conditions, activity, cardiovascular parameters, markers of hypoxia, muscle pathology, and final meat quality in the same birds over time are still scarce; therefore, many proposed links between welfare-related exposures and breast myopathies remain biologically plausible but only partly supported by direct evidence.

## 14. Nutritional Modulation of Breast Muscle Myopathies and Growth-Related Metabolic Stress

Nutritional interventions have been proposed as a means of modifying susceptibility to growth-related breast muscle myopathies, but the available evidence is heterogeneous and does not support a uniform protective effect. Arginine is of particular interest because of its role in nitric oxide synthesis, vascular regulation, and creatine metabolism. In Ross 308 broilers, increasing the digestible Arg:Lys ratio from approximately 1.05–1.07 to 1.15–1.17 improved feed conversion and increased body weight at 33 d from 1829 to 1884 g, but did not significantly reduce the incidence or severity of breast myopathies [69]. Conversely, another study reported that further elevation of the Arg:Lys ratio to approximately 1.35–1.37 reduced the incidence and severity of white striping and spaghetti meat, although wooden breast was unaffected, suggesting that the response depends on dose and myopathy phenotype rather than representing a general protective effect of arginine. Moreover, under hypoxic conditions, supplementation with 0.25% arginine did not consistently protect against myopathy development, illustrating that increased amino acid supply may even intensify metabolic demand when oxygen availability is constrained [70].

Guanidinoacetic acid (GAA), a precursor of creatine, appears more consistently associated with reductions in myopathy severity, although the magnitude of the response remains moderate. In broilers supplemented with 0.06% or 0.12% GAA, the proportion of severe WB decreased from 28.6% in controls to 17.9% and 8.9%, respectively, while the proportion of normal breasts increased from 37.5% to 46.4% and 44.6% [71]. However, moderate WB was more frequent in the 0.12% GAA group, indicating that supplementation may shift severity distribution rather than eliminate the disorder. Commercial-scale data are also inconsistent: reductions in WB incidence of 17–31% have been reported in some trials, whereas benefits were absent at later slaughter ages, emphasizing the importance of age and production context.

Phytase supplementation provides another example of a statistically significant but biologically modest effect. Cauble et al. [72] reported only about a 5% reduction in WB severity, although phytase at 1000–2000 FTU/kg significantly decreased saturated fatty acids and increased PUFA in breast muscle. Thus, the observed benefit may reflect altered lipid metabolism rather than a direct correction of the primary myopathic process.

Antioxidant strategies also show partial efficacy. Wang et al. [73] found that increasing dietary vitamin E to 200 IU/kg reduced WB severity without impairing final body weight or breast yield, and supplementation during the grower phase increased the proportion of unaffected muscle by 16.19% compared with controls. Similarly, combinations of ethoxyquin, organic trace minerals, and organic selenium reduced severe WB incidence under oxidized-fat or heat-stress conditions, but these effects were demonstrated mainly under experimentally induced oxidative challenge rather than standard commercial conditions. Overall, nutritional interventions should therefore be considered modifiers of risk and severity rather than proven preventive strategies. Their effects depend on dose, age, genotype, environmental stress, and the specific myopathy assessed, and the current evidence does not support a single nutritional mechanism capable of consistently preventing WB, WS, and SM.

## 15. Can Meat Quality Defects Be Considered Indicators of Welfare?

Breast muscle myopathies such as wooden breast, white striping, and spaghetti meat are associated with fiber degeneration, necrosis, chronic inflammation, lipidosis, hypoxia, and fibrosis. These lesions reflect prolonged disruption of homeostasis and metabolic overload and may therefore be considered post-mortem markers of abnormal physiological functioning. This does not mean, however, that every meat quality defect is an unequivocal and independent indicator of compromised welfare. Some abnormalities are detected only after slaughter, and their relationships with pain, discomfort, reduced mobility, or behavioral alterations remain incompletely understood. Moreover, individual myopathies may differ in etiology, developmental dynamics, and clinical significance despite partially overlapping histopathological features.

The housing system itself should likewise not be treated as a direct measure of welfare. The source material emphasizes that high production performance does not necessarily indicate good animal welfare and that behavioral expression, activity, leg health, and the opportunity to fulfill natural needs provide important complementary information. Free-range and enriched systems may increase activity and the frequency of natural behaviors, but may also expose birds to infections, parasites, and adverse weather conditions [60,61]. Myopathies should therefore be regarded as one component of a multidimensional welfare assessment together with behavioral measures, gait, mortality, cardiac status, inflammatory markers, and housing conditions. Such an approach reduces the risk of equating every technological abnormality of meat with animal suffering in the absence of functional evidence [2,5,17,27,44].

Importantly, the current evidence does not establish a direct quantitative relationship between the severity of breast myopathies and validated welfare outcomes in the same birds. Most studies characterize lesions post-mortem or focus on production, histopathological, or meat-quality traits, whereas simultaneous measurements of pain-related behavior, locomotor impairment, physiological stress, cardiovascular status, and myopathy severity are scarce. Therefore, WB, WS, and SM should be interpreted as potential retrospective welfare-associated markers rather than validated welfare indicators. Their presence is consistent with chronic physiological disturbance, but the extent to which this disturbance translates into impaired affective state, discomfort, or reduced functional capacity remains insufficiently demonstrated and requires longitudinal studies integrating animal-based welfare measures with ante-mortem physiological assessment and post-mortem muscle pathology [2,5,17,27,44].

The research focus has also shifted over time. Earlier studies emphasized growth rate, feed efficiency, ascites, pulmonary hypertension, and skeletal disorders, whereas more recent work has increasingly addressed breast muscle myopathies, microcirculation, endothelial function, hypoxia, mitochondrial metabolism, and muscle regeneration [4,7,12,21]. This evolution supports an integrated interpretation of the heart, lungs, vasculature, microcirculation, and breast muscle as interacting components of oxygen delivery and utilization, while avoiding the assumption that dysfunction in one component necessarily causes pathology in another.

## 16. Biomarkers of Cardiovascular and Microcirculatory Dysfunction

No single biomarker can reliably characterize cardiovascular and microcirculatory dysfunction in broiler chickens, because the available indicators differ substantially in physiological specificity, timing of measurement, and susceptibility to confounding factors. The right ventricle-to-total ventricle weight ratio (RV:TV) remains one of the most established post-mortem indicators of pulmonary hypertension and right ventricular hypertrophy in broilers [6]. In contrast, ante-mortem measurements such as blood oxygen saturation, hematocrit, blood viscosity, and lactate concentration provide complementary information on systemic oxygen transport and metabolic status. However, these variables are not specific to breast muscle microcirculatory impairment and may also be influenced by age, genotype, environmental conditions, hydration status, handling stress, and the presence of concurrent physiological or pathological challenges [6,7].

Markers of tissue injury may provide additional information but should also be interpreted according to their biological specificity. Cardiac troponins, particularly troponin T, have been investigated as indicators of myocardial injury in broilers; significantly increased serum troponin T concentrations have been reported in hypoxic and ascitic birds, supporting its potential value as an early marker of cardiomyocyte damage [74]. By contrast, creatine kinase (CK) and lactate dehydrogenase (LDH) primarily reflect skeletal muscle cell damage and are therefore not specific indicators of cardiac dysfunction. Importantly, serum CK has shown potential as an ante-mortem biomarker of wooden breast: Kong et al. [75] reported a positive correlation between serum CK activity and breast compression force (r = 0.608; *p* < 0.001), whereas serum LDH showed less consistent discrimination between affected and unaffected birds.

Hypoxia- and angiogenesis-related markers require particularly cautious interpretation. HIF1A transcript abundance should not be considered equivalent to HIF-1α protein accumulation because HIF-1α activity is strongly regulated post-translationally through oxygen-dependent protein stabilization. Thus, increased HIF1A expression does not necessarily demonstrate increased functional HIF-1α signaling [7]. VEGFA, VEGFR-2, angiopoietins, and endothelial markers may provide complementary information on angiogenesis and vascular remodeling, but their expression appears to depend on the stage and severity of muscle pathology [4,7]. In wooden breast, endothelial and vascular alterations have been demonstrated at both molecular and ultrastructural levels, supporting the involvement of endothelial dysfunction in the disease process [4]. Conversely, reduced VEGFA expression reported in severely affected tissue may represent an insufficient or exhausted angiogenic response rather than the absence of hypoxic signaling [4,7].

Consequently, none of these biomarkers should be interpreted in isolation. A multimarker approach combining cardiovascular indices, systemic oxygenation measurements, muscle-injury markers, hypoxia-related signaling, and endothelial/angiogenic indicators is more likely to provide a biologically meaningful assessment of cardiovascular and microcirculatory dysfunction. This is particularly important because central cardiovascular dysfunction, systemic hypoxemia, and local *Pectoralis major* hypoxia represent distinct, although potentially interacting, physiological processes. At present, the strongest evidence supports the use of RV:TV as an indicator of pulmonary hypertension [6], whereas circulating CK, cardiac troponins, HIF-1α-related markers, VEGFA/VEGFR-2, and endothelial markers should largely be considered complementary or exploratory indicators whose predictive value for breast myopathies still requires further validation [4,7,75]. The principal biomarkers, their biological interpretation, ante- or post-mortem applicability, specificity, major confounders, and current strength of evidence are summarized in Table 3.

## 17. Emerging Research Methods

Several emerging technologies have already been applied experimentally to characterize cardiovascular dysfunction or breast muscle myopathies in broilers, whereas others remain largely prospective tools. Echocardiography and Doppler imaging have been used in broilers with ascites and pulmonary hypertension to evaluate cardiac chamber geometry, myocardial function, and blood-flow abnormalities. In ascitic birds, left ventricular fractional shortening was reduced to approximately 21.7%, compared with 39.1% in unaffected broilers and 43.3% in Leghorns, indicating that echocardiography can detect functional cardiac impairment in vivo [23]. However, these techniques have not been validated as predictors of WB, WS, or SM and should therefore be regarded primarily as research tools for cardiovascular phenotyping rather than established diagnostic methods for breast myopathies.

Ultrasound imaging has stronger evidence for direct application to WB detection. Serial ultrasonographic examination of the *Pectoralis major* in live Cobb 500 broilers has been associated with post-mortem WB severity, supporting its potential for ante-mortem identification of affected birds [79]. Nevertheless, validation across different genotypes, ages, and commercial conditions remains limited. Histomorphometry, immunohistochemistry, and electron microscopy provide complementary information on vascular and microstructural abnormalities. For example, Sihvo et al. [13] demonstrated altered pectoral vessel density and early ultrastructural changes in WB, whereas Abasht et al. [4] combined gene-expression analysis, ultrastructural evaluation, and supervised machine-learning methods to demonstrate endothelial alterations associated with WB.

Omics approaches currently provide stronger mechanistic than predictive evidence. Transcriptomic studies have identified early alterations in pathways related to hypoxia, inflammation, extracellular-matrix remodeling, and muscle regeneration [1,3]. Integrative transcriptomic and metabolomic analyses further demonstrated disturbances in energy metabolism and mitochondrial function in WB [22], while circulating metabolite profiles have identified candidate biomarkers associated with WB and WS [80]. However, these approaches largely describe molecular signatures of established or developing pathology and do not yet establish whether the identified markers can reliably predict myopathy before lesions become detectable.

Spectroscopic and computer-vision approaches are also promising, but their current validation is directed mainly toward post-mortem classification rather than ante-mortem prediction. Near-infrared spectroscopy and image-analysis algorithms have achieved high classification accuracy for distinguishing WB from normal fillets [81], supporting their potential use for automated processing-line sorting. These results, however, should not be interpreted as evidence that such methods can detect the underlying cardiovascular or microcirculatory dysfunction before myopathy develops.

Precision-livestock-farming technologies based on sensors, image analysis, microphones, and artificial intelligence should currently be regarded mainly as future integrative tools. Their application in broiler production is better established for monitoring activity, gait, thermal stress, environmental conditions, and general welfare than for predicting WB, WS, SM, or cardiovascular dysfunction. Therefore, the most informative future studies would combine longitudinal sensor data with ante-mortem imaging, circulating biomarkers, vascular and muscle histopathology, and meat-quality measurements in the same birds. Such designs would enable differentiation between markers that precede pathology and those that merely reflect established disease. Figure 1 integrates these emerging approaches within the broader mechanistic framework of cardiovascular function, welfare-related factors, breast muscle myopathies, and meat quality, while highlighting which links are supported by current evidence and which remain hypothetical.

Taken together, the available evidence supports a working model in which rapid muscle hypertrophy increases oxygen and nutrient demand, while insufficient microvascular or cardiorespiratory adaptation could contribute to local perfusion disturbances, hypoxia, mitochondrial dysfunction, oxidative stress, inflammation, fiber degeneration, and fibrosis. However, the strength of evidence differs across individual links in this pathway. Associations between rapid growth, breast yield, and myopathy occurrence are relatively well documented, whereas the causal contribution of central cardiovascular dysfunction to WB, WS, or SM remains insufficiently demonstrated. The model should therefore be regarded as an integrative hypothesis rather than a proven causal sequence.

## 18. Conclusions

Modern broiler breast muscle myopathies arise within a complex biological context shaped by intensive selection for rapid growth and high breast yield, altered muscle architecture, metabolic demand, microvascular adaptation, environmental conditions, and pre-slaughter challenges. The available evidence strongly supports associations among rapid muscle growth, reduced relative capillary supply, mitochondrial and metabolic disturbances, oxidative stress, inflammation, and the development of WB, WS, and SM. However, these relationships should not be interpreted as a single proven causal pathway. In particular, direct evidence linking central cardiovascular dysfunction or pulmonary hypertension with the subsequent development of breast myopathies in the same birds remains limited.

The current literature is constrained by the predominance of cross-sectional and post-mortem studies, heterogeneous definitions and scoring systems for myopathies, differences in genotype, sex, age, slaughter weight, nutrition, and environmental conditions, and the limited number of longitudinal studies under commercial conditions [12,44]. Comparisons between fast- and slow-growing strains are additionally complicated when birds are evaluated at different ages or body weights, making it difficult to separate the effects of growth rate from tissue maturity. Standardized approaches to the assessment of vascular density, tissue hypoxia, endothelial dysfunction, and muscle pathology are therefore needed.

Future research should prioritize longitudinal studies in which cardiovascular function, systemic oxygenation, microcirculation, environmental exposures, activity, circulating biomarkers, muscle histopathology, and final meat quality are evaluated in the same individuals from early growth to slaughter. Such designs are necessary to determine temporal sequence and distinguish primary mechanisms from secondary consequences. Nutritional and management interventions should also be tested within this integrated framework rather than as isolated treatments. From a production perspective, the challenge is not simply to maximize growth further, but to achieve a sustainable balance among muscle growth potential, cardiovascular and metabolic capacity, bird welfare, and final product quality [1,2,3,4,7,12,13,14,21,22].

## Figures and Tables

**Table 1:** Comparative pathophysiological characteristics and quantitative meat-quality consequences of major growth-related breast muscle myopathies in broiler chickens.

Myopathy	Macroscopic Characteristics	Main Physiological/Histopathological Features	Vascular/Metabolic Involvement and Strength of Evidence	Effects on Meat Quality	Representative Quantitative Evidence	Key References
Wooden breast (WB)	Palpable hardness and rigidity of the Pectoralis major; pale and bulging areas; increased fillet thickness; frequently co-occurs with WS.	Myofiber degeneration and necrosis; marked variation in fiber size; inflammatory infiltration and regenerative changes; interstitial edema; lipidosis; extensive fibrosis and increased collagen deposition. Phlebitis, perivascular lesions, and endothelial alterations have also been described.	Relatively strong evidence of association with impaired microvascular function, including reduced vascular supply, vascular/endothelial lesions, hypoxia-related signaling, oxidative stress, and metabolic disturbance. However, whether vascular impairment is a primary event or partly secondary to established tissue remodeling remains unresolved.	Usually the most pronounced deterioration in technological quality: higher ultimate pH and cooking loss; reduced water-holding/binding capacity and processing yield; increased raw hardness and, particularly in severe WB, increased cooked shear force; altered protein, fat, collagen, and water distribution.	Bian et al. (2024): normal → severe WB: pH 5.91 → 6.00 (*p* = 0.047); cooking loss 14.31 → 21.58% (*p* < 0.001); shear force 32.78 → 45.10 N (*p* = 0.003); hardness 2814.96 → 3576.05 g (*p* = 0.019) [32].	[2,13,14,28,29,32,33,34,35]
White striping (WS)	White striations parallel to muscle fibers, ranging from thin superficial lines to broad white bands; often co-occurs with WB.	Myofiber degeneration with variable regeneration; increased interstitial adipose and connective tissue deposition; lipidosis; fibrosis generally less extensive than in severe WB.	Strong association with rapid growth, breast yield, and muscle hypertrophy. Metabolic stress and impaired oxygen delivery may contribute, but direct evidence supporting a primary vascular mechanism is less consistent than for WB.	Generally moderate and severity-dependent effects: higher ultimate pH in severe cases; reduced marinade uptake and processing yield; increased cooking losses; altered water-holding/binding capacity. Texture effects are variable and may differ from WB.	Petracci et al. (2013): normal → severe WS: pH 5.86 → 5.95; marinade uptake 12.67 → 7.92%; cooking loss in raw meat 21.27 → 26.74% (all *p* ≤ 0.05). Commercial WS incidence: 12.0%; high-breast-yield vs. standard hybrids: 15.2 vs. 10.0% (*p* ≤ 0.001) [36].	[2,33,34,35,36,37,38]
Spaghetti meat (SM)	Soft, loose, poorly cohesive breast muscle with separation of fiber bundles, particularly in the superficial/cranial region; fibers can be readily pulled apart into strand-like structures.	Loss of connective-tissue integrity; altered perimysial and endomysial organization; extracellular-matrix remodeling; reduced collagen cross-linking; separation of fiber bundles. Degenerative/regenerative alterations may coexist, but the connective-tissue phenotype differs from WB.	Current evidence supports a prominent role for connective-tissue and extracellular-matrix abnormalities rather than a simple extension of the WB hypoxia/vascular model. Hypoxia or metabolic stress may contribute, but direct causal evidence is less developed.	Reduced structural cohesiveness and softer cooked texture; altered water distribution and composition may occur. Effects on pH and water-holding properties are less consistent across studies and are strongly influenced by coexistence with WB and/or WS.	Baldi et al. (2019): SM showed significantly lower collagen cross-linking and softer cooked texture than unaffected or other abnormality groups (*p* < 0.001). Quantitative pH/WHC effects are less consistent and should not be presented as universal diagnostic values [33].	[2,33,34,35,39,40]

Abbreviations: WB, wooden breast; WS, white striping; SM, spaghetti meat. Arrows indicate the direction of change from unaffected/normal to severe myopathy. Numerical values are representative of individual studies and should not be interpreted as universal thresholds, because outcomes vary with genotype, slaughter age and weight, myopathy severity, coexistence of abnormalities, sampling location, and analytical methodology.

**Table 2:** Representative evidence linking welfare-related environmental and management factors with physiological responses and meat-quality outcomes in broiler chickens.

Factor	Representative Experimental Conditions	Main Physiological/Welfare-Related Response	Reported Meat-quality Outcome	Selected Quantitative Evidence	Critical Interpretation/Limitation	Key References
Heat stress	Cyclic or chronic exposure to elevated ambient temperature; representative study: 32.5 ± 1.4 °C for 8 h/day vs. 21.3 ± 1.2 °C.	Panting and CO_2_ loss; acid–base disturbance; redistribution of blood flow for heat dissipation; increased oxidative stress and mitochondrial dysfunction; reduced feed intake and altered energy metabolism.	Lower early post-mortem pH; increased drip loss and, in some studies, cooking loss and lightness; reduced water-holding capacity. Effects depend strongly on intensity and duration of heat exposure and timing relative to slaughter.	Heat-stressed vs. thermoneutral breast muscle: pH45 min 6.68 vs. 6.84 (*p* = 0.035); drip loss day 1, 3.74 vs. 0.90%; day 2, 4.81 vs. 1.96%; day 3, 5.53 vs. 2.98% (*p* ≤ 0.003). Cooking loss: 13.79 vs. 10.56% (NS).	Evidence for direct effects of heat stress on redox status and post-mortem metabolism is relatively strong. However, chronic rearing heat stress and acute pre-slaughter heat exposure should be considered separately because they may affect meat quality through different temporal mechanisms.	[24,25,51,64,65]
Stocking density	Examples include 29 vs. 37 kg/m^2^, or graded densities of 16–26 birds/m^2^ under warm conditions; density effects are often accompanied by changes in litter, heat load, air quality, and activity.	Reduced locomotor space and activity; increased disturbance of resting birds; potentially greater heat and respiratory load when microclimate deteriorates; increased risk of footpad and leg lesions at unfavorable conditions.	Effects on pH, water-holding capacity, cooking loss, drip loss, and shear force are inconsistent among studies and often interact with genotype and environmental temperature.	At 29 vs. 37 kg/m^2^, conventional broilers showed breast cooking loss 8.79 vs. 9.85%, while slow-growing birds showed 7.14 vs. 8.40%; strain × density interaction affected 24 h pH (*p* = 0.03). Under high temperature, WHC ranged from 49.4% at 16 birds/m^2^ to 54.1% at 23 birds/m^2^ (*p* = 0.010), whereas pH and cooking loss were unchanged.	Stocking density should not be treated as an isolated causal factor. Several studies indicate that genotype and accompanying microclimate may explain more variation than bird numbers per se; therefore, density should be interpreted together with temperature, ventilation, litter condition, and growth rate.	[27,55,56,57,58,59,66,67]
Lighting program/dark period	Continuous or near-continuous light compared with intermittent lighting or graded photoperiods (14L:10D, 17L:7D, 20L:4D, 23L:1D).	Dark periods support melatonin rhythms, rest and recovery. Intermittent lighting increased antioxidant defenses; longer day length was associated with poorer gait/footpad outcomes and greater mortality from metabolic/skeletal causes.	Direct evidence linking photoperiod to breast-meat pH, WHC, or myopathy incidence is limited. Most available evidence supports indirect effects through activity, rest, metabolic status, antioxidant capacity, and health.	Compared with 24L:0D, intermittent lighting significantly increased serum melatonin, total antioxidant capacity, superoxide dismutase and glutathione peroxidase activities, and reduced hepatic malondialdehyde (*p* < 0.05 in reported comparisons). Mortality and painful gait categories increased with longer day length in graded-photoperiod studies.	Evidence is stronger for welfare/physiological outcomes than for direct meat-quality effects. The manuscript should therefore present lighting primarily as a risk-modifying exposure rather than as an established direct cause of breast myopathy or meat-quality defects.	[30,31,48,49,50]
Housing system/environmental enrichment	Indoor litter or slatted-floor systems compared with free-range/outdoor access and/or enriched environments; effects depend strongly on genotype and use of the outdoor area.	Greater opportunity for locomotion and species-specific behaviors; potential improvement in leg and bone condition, but also greater exposure to weather variability, pathogens, and parasites. Activity response is genotype-dependent.	Housing can modify color, water retention, texture, fat content, and in some studies myopathy prevalence, but genotype frequently exerts an equal or stronger effect.	In slow-growing Hubbard broilers, housing significantly affected b* and chroma (*p* = 0.0001), cooking loss and some texture traits; spaghetti meat was more frequent indoors than in free-range birds (*p* = 0.01), whereas WB and WS were not significantly affected by housing.	Housing labels (indoor, free-range, enriched) are not direct measures of welfare. Effects on meat quality should be interpreted with genotype, activity level, climate exposure, diet, slaughter age, and actual range use. Direct cardiovascular measurements are scarce.	[60,61,62,63,68]
Transport and pre-slaughter stress	Short vs. prolonged transport and different recovery/lairage periods; representative design: 45 min or 3 h transport followed by 45 min or 3 h recovery.	Acute activation of the stress response; altered plasma corticosterone and glucose; glycogen depletion; increased glycolysis/lipolysis; temperature and ventilation during transport modify the response.	May alter post-mortem glycolysis, pH development, color, and water-holding capacity. Effects can occur independently of chronic microvascular mechanisms that develop during rearing.	Three-hour transport reduced breast-muscle glycogen (trend, *p* = 0.06) and altered muscle-fiber characteristics; recovery duration affected corticosterone (*p* = 0.05), thigh glycogen (*p* < 0.05), lactate and glycolytic potential (both *p* < 0.01). Longer recovery reduced metabolic disturbance.	This is an acute pre-slaughter pathway and should be separated conceptually from chronic growth-related myopathy mechanisms. Transport effects depend on duration, thermal conditions, crate density, ventilation, handling, and recovery time.	[46,47]

b* = yellowness coordinate in the CIELAB color space; Abbreviations: WHC, water-holding capacity; WB, wooden breast; WS, white striping; NS, not significant. Numerical values are representative study-specific results rather than universal thresholds. Environmental and management exposures often co-occur, and causal interpretation should account for genotype, age, growth rate, diet, microclimate, and pre-slaughter conditions.

**Table 3:** Potential biomarkers of cardiovascular and microcirculatory dysfunction in broiler chickens: biological meaning, sampling window, specificity, major confounders, and current strength of evidence.

Category	Biomarker	What It Measures/Biological Meaning	Ante-/Post-Mortem Applicability	Specificity	Major Confounders/Interpretive Limitations	Strength of Evidence and Key Support
Cardiovascular morphology	RV:TV ratio	Degree of right ventricular hypertrophy associated with chronically increased pulmonary vascular resistance and pulmonary hypertension.	Post-mortem only.	High for pulmonary hypertension/ascites phenotype; low for local breast-muscle microcirculatory dysfunction.	Age, genotype, altitude, cold exposure, chronicity of pulmonary hypertension, cardiac remodeling. Does not directly quantify pectoral perfusion or local hypoxia.	Strong/established for pulmonary hypertension syndrome [6]
Systemic oxygen transport	Hematocrit	Packed-cell volume and erythrocyte concentration; may increase as an adaptive response to chronic hypoxemia.	Ante-mortem blood sample.	Moderate for chronic systemic oxygenation stress; not specific for cardiovascular disease or breast myopathy.	Hydration/dehydration, age, sex, altitude, thermal stress, disease status, sampling conditions. Elevated hematocrit does not prove local muscle hypoxia.	Moderate/supportive [6]
Systemic oxygenation	SpO_2_/hemoglobin O_2_ saturation	Peripheral/systemic blood oxygenation and efficiency of pulmonary oxygen transfer.	Ante-mortem, potentially repeated/non-lethal measurement.	Moderate for systemic hypoxemia; low for local Pectoralis major hypoxia.	Probe site and contact, motion, peripheral perfusion, temperature, handling stress, device calibration; normal SpO2 does not exclude local microvascular hypoxia.	Moderate/physiologically relevant, but interpretation for breast myopathy requires caution [6]
Metabolic/perfusion-related	Lactate	Balance between lactate production, tissue perfusion, clearance, and anaerobic glycolytic activity.	Ante-mortem blood; tissue lactate can be measured post-mortem.	Low; reflects metabolic stress but is not specific for cardiac or microvascular dysfunction.	Handling, exercise/wing flapping, heat stress, transport, feed status, sampling time, post-mortem glycolysis.	Exploratory/supportive. Best interpreted with oxygenation, perfusion and histopathology rather than alone. Altered lactate metabolism and reduced lactate accumulation have been reported in WB muscle [76,77]
Cardiac injury	Cardiac troponins (e.g., cTnT/cTnI)	Release from injured cardiomyocytes; reflects myocardial cell injury rather than hemodynamic dysfunction per se.	Ante-mortem serum/plasma; repeatable in principle.	Relatively high for myocardial injury, but not a direct marker of pulmonary hypertension or breast-muscle perfusion.	Assay validation in chickens, age, hypoxia, systemic illness, severity/stage of cardiac injury.	Moderate for myocardial injury; limited validation as a routine broiler selection/myopathy-risk marker [74]
Skeletal muscle injury	Creatine kinase (CK)	Leakage of muscle enzyme after sarcolemmal/myofiber damage; can reflect severity of breast-muscle injury.	Ante-mortem serum/plasma; muscle activity post-mortem can also be measured.	Moderate for skeletal-muscle damage; low for cardiac specificity and microvascular cause.	Handling, muscle exertion, injections/trauma, other myopathies, genotype, age, assay units and laboratory platform.	Moderate and promising for WB. Serum CK increased with WB severity and correlated with compression force (r = 0.608, *p* < 0.001) [75]
Tissue injury/metabolism	Lactate dehydrogenase (LDH)	Cellular injury and altered lactate-pyruvate metabolism; may reflect muscle damage and metabolic disturbance.	Ante-mortem serum/plasma; tissue enzyme activity post-mortem.	Low; widely distributed enzyme and not specific for heart, endothelium, or WB.	Hemolysis, liver injury, skeletal-muscle injury, heat stress, oxidative status, assay method; serum and tissue LDH may show different patterns.	Exploratory/low-moderate. Useful only as part of a marker panel, not as a stand-alone cardiovascular biomarker. Serum LDH was higher in severe WS than in normal birds (7024.71 vs. 3101.67 U/L; *p* = 0.01), and WB muscle shows marked alterations in LDH isoenzyme expression/activity [76,78]
Hypoxia signaling	HIF-1α/HIF1A	Hypoxia-responsive transcriptional signaling. HIF-1α protein stabilization reflects oxygen-dependent pathway activation; HIF1A transcript abundance is not equivalent to HIF-1α protein accumulation.	Primarily post-mortem tissue (protein, transcript, immunohistochemistry); limited practical ante-mortem use.	Moderate for hypoxia-related signaling; not specific for cardiovascular origin.	Disease stage, sampling location, time from sampling to preservation, protein vs. mRNA endpoint, inflammation and metabolic stress.	Moderate mechanistic evidence [7]
Angiogenesis/vascular adaptation	VEGFA/VEGFR-2	Angiogenic signaling and vascular adaptation to tissue demand/hypoxia; VEGFR-2 reflects a major endothelial VEGF signaling axis.	Mostly post-mortem tissue expression/protein assays.	Moderate for angiogenic response; not specific for the initiating cause of myopathy.	Age, genotype, disease stage and severity, anatomical sampling site, transcript vs. protein measurement. Reduced VEGFA in severe lesions may reflect failure/exhaustion rather than absence of hypoxia.	Moderate but stage-dependent [4,7]
Endothelial dysfunction/vascular remodeling	Endothelial markers (e.g., ESM1/endocan) and endothelial ultrastructure	Endothelial activation/dysfunction, altered vascular-cell metabolism, and structural remodeling of small vessels. ESM1 expression increased with WB severity in one study; ultrastructural changes indicate an activated endothelial phenotype.	Predominantly post-mortem muscle tissue (gene expression, immunohistochemistry, electron microscopy). Circulating validation is not yet established for routine use.	Moderate for local endothelial dysfunction in WB; limited generalizability across all myopathies.	Disease stage, lesion severity, sampling location, vascular compartment, inflammation, lipid infiltration, method-specific variability.	Moderate/emerging. Abasht et al. [4] provide molecular and ultrastructural evidence of endothelial dysfunction in WB.

Abbreviations: RV:TV, right ventricular weight to total ventricular weight ratio; SpO_2_, peripheral oxygen saturation; CK, creatine kinase; LDH, lactate dehydrogenase; HIF-1α, hypoxia-inducible factor 1 alpha; VEGFA, vascular endothelial growth factor A; VEGFR-2, vascular endothelial growth factor receptor 2; ESM1, endothelial cell-specific molecule 1. Evidence levels are qualitative and refer to the degree of validation in broiler cardiovascular physiology and/or breast-muscle myopathy research, not to clinical diagnostic cut-offs.

## Data Availability

No new data were created or analyzed in this study. Data sharing is not applicable to this article.
